# Comparative Analysis of Proteomes and Functionomes Provides Insights into Origins of Cellular Diversification

**DOI:** 10.1155/2013/648746

**Published:** 2013-12-31

**Authors:** Arshan Nasir, Gustavo Caetano-Anollés

**Affiliations:** Evolutionary Bioinformatics Laboratory, Department of Crop Sciences, and Illinois Informatics Institute, University of Illinois Urbana-Champaign, Urbana, IL 61801, USA

## Abstract

Reconstructing the evolutionary history of modern species is a difficult problem complicated by the conceptual and technical limitations of phylogenetic tree building methods. Here, we propose a comparative proteomic and functionomic inferential framework for genome evolution that allows resolving the tripartite division of cells and sketching their history. Evolutionary inferences were derived from the spread of conserved molecular features, such as molecular structures and functions, in the proteomes and functionomes of contemporary organisms. Patterns of use and reuse of these traits yielded significant insights into the origins of cellular diversification. Results uncovered an unprecedented strong evolutionary association between Bacteria and Eukarya while revealing marked evolutionary reductive tendencies in the archaeal genomic repertoires. The effects of nonvertical evolutionary processes (e.g., HGT, convergent evolution) were found to be limited while reductive evolution and molecular innovation appeared to be prevalent during the evolution of cells. Our study revealed a strong vertical trace in the history of proteins and associated molecular functions, which was reliably recovered using the comparative genomics approach. The trace supported the existence of a stem line of descent and the very early appearance of Archaea as a diversified superkingdom, but failed to uncover a hidden canonical pattern in which Bacteria was the first superkingdom to deploy superkingdom-specific structures and functions.

## 1. Introduction

Tracing the evolution of extant organisms to a common universal cellular ancestor of life is of fundamental biological importance. Modern organisms can be classified into three primary cellular superkingdoms, Archaea, Bacteria, and Eukarya [1]. Molecular, biochemical, and morphological lines of evidence support this trichotomous division. While the three-superkingdom system is well accepted, establishing which of the three is the most ancient remains problematic. Initial construction of unrooted phylogenies based on the joint evolution of genes linked by an ancient gene duplication event revealed that, for each set of paralogous genes, Archaea and Eukarya were sister groups and diverged from a last archaeal-eukaryal common ancestor [2, 3]. This “canonical” rooting that places Bacteria at the base of the “Tree of Life” (ToL) is still widely accepted despite the fact that many other paralogous gene couples produced discordant topologies and despite known technical artifacts associated with these sequence-based evolutionarily deep phylogenies [4, 5]. As a result, reconstructing a truly “universal” ToL portraying the evolutionary relationships of all existing species remains one of the most controversial issues in evolutionary biology. This in part owes to the shortcomings of available phylogenetic characters and tree optimization methods that suffer from important technical and conceptual limitations [6, 7] and have failed to generate a consensus. It is further complicated by the fact that genetic material can be readily exchanged between species, especially akaryotes (i.e., Archaea and Bacteria that lack a nucleus) via horizontal gene transfer (HGT) [8–10]. Nonvertical evolutionary processes coupled with uncertainties regarding evolutionary assumptions greatly complicate the problem of reconstructing the evolutionary past. Recently, ToLs reconstructed using conserved structural information of protein domains [11, 12], their annotated functions (Kim et al., ms. resubmitted), and universal RNA families [13–18] provided new ways to root phylogenies. These studies identified thermophilic archaeal species to be the most closely related to the primordial cells. Findings not only challenge the bacterial rooting of the ToL but also highlight the importance of employing reliable phylogenetic methods and assumptions when reconstructing deep evolutionary history [7].

Here, we advance the structural and functional approach by providing a simple solution to the problem of phylogenetic reconstruction. We argue that basic quantitative and comparative genomic analyses that do not invoke phylogenetic reconstruction are sufficient to resolve the tripartite division of cells and sketch their history. Our comparative approach involves the analysis of how superkingdoms, and their organismal constituents, relate to each other in terms of global sharing of genomic features. The genomic features we selected are entire repertoires of molecular structures and functions (collectively referred to as traits from hereinafter). They define two specific genomic datasets. The *structure* dataset encompasses the occurrence and abundance of 1,733 fold superfamily (FSF) domains in 981 completely sequenced proteomes. FSF domains were delimited using the Structural Classification of Proteins (SCOP ver. 1.75), which is a manually curated database of structural and evolutionary information of protein domains [19, 20]. The FSF level of the SCOP hierarchy includes domains that have diverged from a common ancestor and are evolutionarily conserved [21, 22]. In comparison, the *function* dataset describes the occurrence and abundance of 1,924 gene ontology (GO) terms [23, 24] in 249 functionomes. We note that the global set of FSFs portrays the entire structural repertoire of organisms and that the repertoire of GO terms portrays their true physiology. Both provide useful information about species diversification. We restricted our analyses to include only structures and functions as they are more conserved than gene sequences [25–27] and permit deep evolutionary comparisons. In contrast, nucleotide sequences are susceptible to higher mutation rates and are continuously rearranged in genomes to yield novel domain combinations and molecular functions [6]. In other words, loss of an FSF domain structure or molecular function is much more costly for cells as it sometimes involves loss of hundreds of genes that have accumulated over long periods of evolutionary time. This is compounded especially for traits that are very ancient as they had more time to multiply in genomes and increase their genomic abundance [28, 29]. Thus molecular structure and function remain preserved in cells for relatively longer periods and make reliable candidates for inferring deep evolutionary relationships.

**Figure 1:**
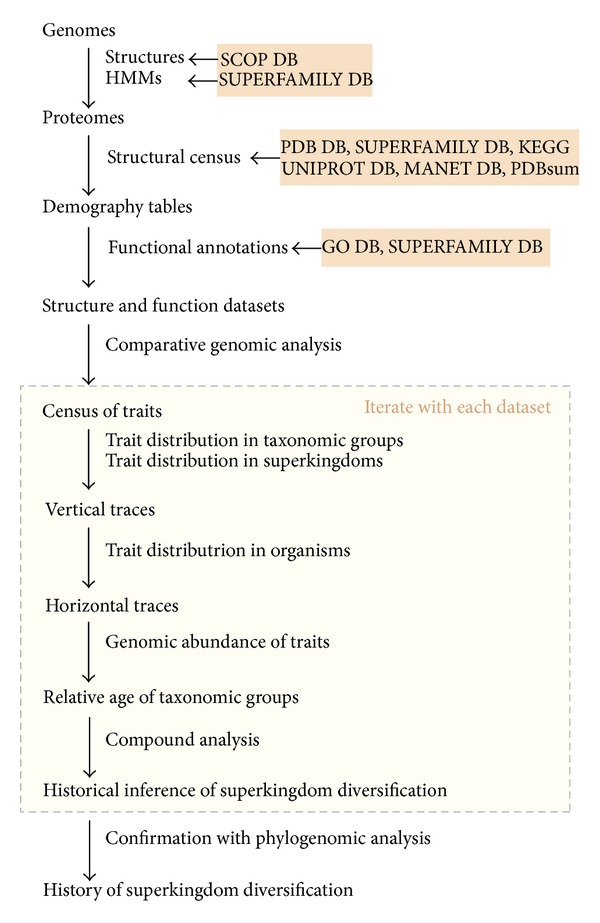
Overview of the comparative proteomics and functionomics methodology. Proteomes and functionomes were scanned for the occurrence and abundance of FSFs and GO terms (i.e., traits). This information was represented in data matrices that were analyzed for trends of trait sharing and traces of vertical and horizontal inheritance. Inferences were drawn regarding superkingdom diversification and were confirmed with previously published phylogenetic studies.

Here, we show that an analysis of trait distribution between superkingdoms, distributions between genomic repertoires of superkingdoms, and abundance counts allow dissection of historical (ideographic) patterns using a comparative ahistorical (nomothetic) method (Figure 1). Inspired by a comparative analysis of RNA families [30], we measured the strength of evolutionary association between superkingdoms as a function of patterns of sharing of individual traits (Figure 1). We note that our approach is sufficiently informative to make reliable inferences regarding different evolutionary scenarios of diversification adopted by the three superkingdoms. This approach revisits widely accepted theories regarding the origin of diversified life [31, 32] and falsifies the fusion [33] and hydrogen hypotheses [34] of eukaryotic origins, more than supporting any. This exercise then prompts validation by phylogenetic tree reconstruction, which we have reported previously (see [26, 28, 29, 35]). In light of these considerations, the comparative exercise provides an easy-to use and reliable alternative to otherwise complicated phylogenetic tree reconstruction methods. These analyses carry the potential to yield significant insights into the evolution of cells and, if carefully interpreted, provide strong arguments in favor of the rooting of the ToL in Archaea and embedded canonical pattern of FSF and GO innovation.

## 2. Materials and Methods

### 2.1. Data Retrieval and Manipulation

FSF domain assignments for 981 completely sequenced proteomes were extracted from local MySQL installation of SUPERFAMILY ver. 1.75 database [36] using a stringent *E*-value cutoff of 10^−4^ [37]. The SUPERFAMILY database assigns structures to protein sequences using profile hidden Markov models (HMMs) searches that are superior in detecting remote homologies [38]. The dataset included 652 bacterial, 70 archaeal, and 259 eukaryal proteomes encoding a total repertoire of 1,733 significant FSF domains. In this study, FSFs were identified using SCOP alphanumeric identifiers (e.g., c.37.1, where c represent the class of domain structure (*α*, *β*, *α* + *β*, *α*/*β*, etc.), 37 the fold, and 1 the FSF). This constituted the *structure* dataset.

To prepare the *function* dataset, we downloaded the Gene Ontology Association (GOA) files for 1,595 organisms from the European Bioinformatics Institute (http://www.ebi.ac.uk/GOA/proteomes). These files were filtered to exclude strain-level and parasitic organisms. They were subjected to a 50% GO coverage threshold (number of gene products annotated to GO terms divided by the total number of gene products) to ensure high quality annotations. In this study, we only sampled terminal-level GO terms from the GO molecular function hierarchy (simply referred to as GOs or functions from hereinafter), as they represent the highly-specialized functional annotations and approximate the molecular activities of cells (which are evolutionarily informative) [25]. We further excluded GOs that were likely candidates of HGT by scanning the total set of 2,039 terminal GOs in our dataset against proteins listed in the horizontal gene transfer database (HGT-DB) [39]. This allowed the exclusion of 115 potentially HGT-derived GOs. The final *function* dataset included 249 free-living functionomes from 183 Bacteria, 45 Archaea, and 21 Eukarya encoding a total set of 1,924 GOs.

### 2.2. Genomic Census of Traits

We conducted a genomic census for both *structure* and *function* datasets by counting the occurrence (presence/absence) and abundance (redundant counts) of traits in all proteomes and functionomes. These data matrices were then scanned to generate Venn diagrams and boxplots displaying patterns of trait sharing both between and within proteomes and functionomes of superkingdom groups.

### 2.3. Calculating the Spread of Traits in Proteomes and Functionomes

The spread of each trait in a superkingdom was calculated by an *f*-value indicating the number of proteomes/functionomes harboring a trait divided by the total number of proteomes/functionomes in that organismal group. The *f*-value approaches one for ubiquitous traits but is lower for those that are less widely distributed.

### 2.4. Estimating the Evolutionary Age of Traits

We used a relative time scale to pinpoint the origin of FSFs in molecular evolution. This scale was defined by node distance (*nd*) as calculated from a phylogenetic tree of FSF domains (see [26, 28, 35] for practical details). Technically, *nd* is the distance of a particular trait from its position on the phylogenetic tree to the root node. It is given on a scale from 0 (the most ancient or root node) to 1 (highly derived or terminal node). Biologically, it reflects the evolutionary age of an FSF relative to other FSFs. *nd* has been successfully used in the past to describe important events in the evolution of cells (e.g., [26, 28]) and could be considered a reliable proxy to estimate the origin of molecular traits in organisms.

## 3. Results

### 3.1. Identifying Vertical Traces

**Figure 2:**
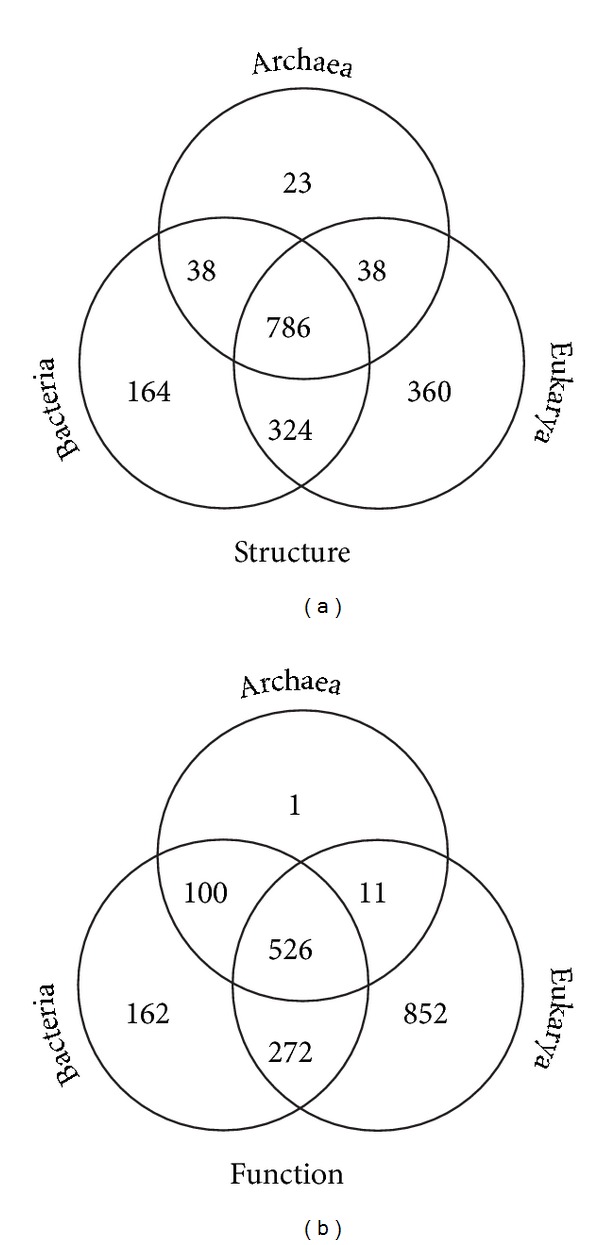
Global trends of trait sharing in Venn taxonomic groups. (a) Venn diagram displaying the distribution of 1,733 FSF domains in 981 completely sequenced proteomes sampled from 652 Bacteria, 70 Archaea, and 259 Eukarya. This constituted the *structure* dataset. (b) Venn diagram displaying the distribution of 1,924 terminal-level GOs in 249 free-living functionomes corresponding to 183 Bacteria, 45 Archaea, and 21 Eukarya. This constituted the *function* dataset.

Venn diagrams demonstrate the evolutionary sorting of FSF and GO traits in the seven possible and mutually exclusive Venn taxonomic groups, ABE (i.e., present in all three superkingdoms), AB (present only in akaryotes), BE (present only in Bacteria and Eukarya), AE (present only in Archaea and Eukarya), and the three superkingdom-specific groups, A, B, and E (Figure 2). Remarkably, a majority of the traits (45% of total structures and 27% of functions) were present in all three superkingdoms, supporting the hypothesis of common ancestry (Figure 2). Since a ToL by definition is a nested hierarchy of taxonomies, we propose that elevated sharing of traits by a taxonomic group points towards an ancient “vertical trace” indicative of divergence from a common ancestor. In turn, low numbers in a taxonomic group are indicative of other evolutionary processes besides lineage splitting, including reductive evolution, HGT, convergent evolution, differential loss, and secondary evolutionary adaptations.

The two-superkingdom taxonomic groups were most informative as each embodied a possible vertical trace and an evolutionary hypothesis of superkingdom origin. The number of traits in the AB, AE, and BE taxonomic groups is therefore indicative of the strength of evolutionary association between akaryotes, Archaea and Eukarya, and Bacteria and Eukarya, respectively. Remarkably, and against intuition, the size of the AB and AE taxonomic groups was ~9 folds smaller than that of BE in the *structure* dataset (38 and 38 versus 324) (Figure 2(a)). This trend was also recovered in the *function* dataset where BE significantly outnumbered both AB and AE (272 versus 100 and 11) (Figure 2(b)). These important biases suggest an intriguing ancestral evolutionary link between Bacteria and Eukarya, very much as the large number of ABE traits suggests an ancestral link between all organisms. While simultaneous gains of traits in both bacterial and eukaryal proteomes would be possible, the high sharing of structures and functions by the BE taxonomic group makes it parsimoniously unlikely and points instead to an evolutionary scenario in which the two superkingdoms diverged from a common ancestor. This is particularly supported by the findings that convergent evolution of structures is rare [40] and seems unlikely to occur at such high levels. We note that bacterial organisms are more intimately associated with eukaryotes, establishing many coevolving bacterial parasitic/symbiotic interactions with eukaryotic hosts; this is in marked contrast with organismal interactions involving Archaea [41]. These interactions could foster the exchange of protein and functional repertoires between the organisms. However, the *function* dataset included only free-living GO-annotated organisms with the exclusion of HGT-acquired GOs and consequently was free from adaptive effects of either parasitic or symbiotic lifestyles. The dataset still showed the high representation of the BE group relative to the AB and AE groups (Figure 2(b)). In short, the very large size difference of BE compared to the AB and AE groups is an evolutionarily significant outcome that cannot be explained merely by parasitic/symbiotic processes.

Finally, the Venn diagrams show that Eukarya-specific traits always outnumbered Bacteria-specific and Archaea-specific counterparts, suggesting either an expansive mode of evolutionary growth of eukaryotic repertoires or a reductive mode in akaryotic counterparts, or both (Figure 2). This is an expected result as eukaryotes encode a highly diverse and complex genome and are capable of carrying out many advanced molecular activities, especially those related to development and immunological responses. Based on our initial comparative genomic exercise, we put forth three preliminary conclusions: (i) all extant cells are related by common descent, (ii) Bacteria and Eukarya diverged from a mutual ancestor, and (iii) eukaryotes are significantly more complex than akaryotes in terms of numbers of unique traits.

### 3.2. Identifying Horizontal Traces

Venn diagrams simply describe global patterns of sharing in superkingdoms and cannot dissect how popular are traits in the organisms of each superkingdom. In other words, the presence of a trait in a superkingdom does not necessarily imply that it was vertically inherited; this trait might only be present in few of its members. In such cases, acquisition of traits by nonvertical (e.g., HGT fluxes, convergent evolution) or confounding (e.g., differential loss that mimics HGT) evolutionary processes becomes more likely. To fully explore the extent to which these real or virtual “horizontal traces” contribute to the development of the proteomes of organisms in superkingdoms and to further test the preliminary conclusions drawn from the Venn diagrams of Figure 2, we calculated the spread or popularity of FSF and GO traits in the organisms of superkingdoms, which we term *f*-value. The *f*-value is simply the number of organisms in a Venn taxonomic group harboring a trait divided by the total number of organisms in that taxonomic group and in that superkingdom. It is given on a relative scale from 0 (absent) to 1 (omnipresent). Using this simplistic approach, we first identified 17 FSFs (Table 1) and 26 GOs (Table 2) that were present in all proteomes and functionomes, respectively. This cohort of traits truly represents the “universal” core of traits that was present in the common ancestor of life, the urancestor, and was strongly retained by all of its descendants. These traits perform crucial and central metabolic and informational roles in cells such as ATP hydrolysis and ion binding, make up structural components of ribosomal proteins, and are involved in DNA replication and protein translational processes (Tables 1 and 2). Moreover, a total of 245 FSFs and 95 GOs had an *f* > 0.90 implying near-universal presence and suggesting reductive losses in the remaining 10% of the proteomes and functionomes (data not shown). This global analysis based on popularity of traits in proteomes and functionomes suggests that the urancestor was especially enriched (structurally and functionally) in metabolic functions [29] and illustrates the power of *f*-value in dissecting traces of vertical versus horizontal inheritance. Therefore, we extended this analysis to the proteomes and functionomes of members of each of the seven taxonomic groups.

**Figure 3:**
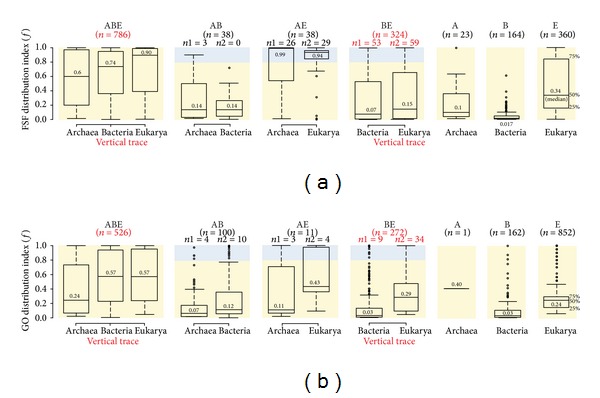
The spread of FSF domain structures (a) and GO terminal terms (b) in the proteomes and functionomes of each member of the superkingdom in the seven Venn taxonomic groups (panels ABE, AB, AE, BE, A, B, and E). Shaded regions indicate that FSFs or GOs were present in >80% of the proteomes (*f* > 0.8), and their numbers, *n*_1_ and *n*_2_. Numbers in boxplots of each distribution indicate group medians. Numbers in red suggest the strongest vertical evolutionary trace.

We first compared the spread of FSFs in the *structure* dataset using boxplot representations of *f*-value distributions (Figure 3(a)). Our assumptions are straightforward: high *f*-values and balanced *f*-distributions reflect vertical traces while low *f*-values and biased *f*-distributions echo horizontal (flux-loss) traces, respectively. The 786 ABE structures were distributed with the highest *f*-values and the medians increased in the order, Archaea (median *f* = 0.6), Bacteria (0.74), and Eukarya (0.90) (Figure 3(a), ABE taxonomic group). The large number of ABE structures that was widespread in all three superkingdoms strengthens the hypothesis of life's common ancestry. The relatively lower median *f*-values in akaryotes (0.6 for Archaea and 0.74 for Bacteria versus 0.90 in Eukarya) can be explained by genome reduction events that are known to occur with relatively high frequency in akaryotic microbes [26, 42], and also manifest in the numbers of superkingdom-specific traits (Figure 2). The 38 AB structures were poorly but similarly distributed (median *f*-values = 0.14) in archaeal and bacterial proteomes, with archaeal structures exhibiting a tendency to become more widespread (longer tail) (Figure 3(a), AB). This pattern supports the existence of a horizontal trace between akaryotes, with a weak bias in flux-loss between superkingdoms (note however that no common outliers could be detected). In contrast, the 38 AE structures were highly represented (median *f*-values > 0.94) in the organisms of corresponding superkingdoms (Figure 3(a), AE). Again, archaeal structures appeared more widely shared but also showed a longer tail indicative of possible flux-loss episodes. At first glance, this chimes for a strong vertical trace of the AE group that could rival that of the BE group. However, this may not be the case. The 324 BE structures were on average poorly represented in bacterial and eukaryal proteomes (median *f*-values < 0.15) (Figure 3(a), BE). Their overall spread was relatively uniform, with a weak bias towards higher representation in Eukarya. However, 53 and 59 structures were widespread in the proteomes of Bacteria and Eukarya (*f* > 0.8), respectively (shaded region in Figure 3(a), BE). This subset of BE structures was numerically double that of the total set of the highly represented AE structures. Thus, the stronger vertical trace for BE structures continues to support a sister-group relationship between Bacteria and Eukarya and the early diversification of Archaea. We note that this inference is strengthened by the fact that we had 652 bacterial and 259 eukaryal proteomes in comparison to only 70 archaeal proteomes. Existence of any structure in such large number of genomes implies strong selective pressure and conservation of that trait. Finally, the sharing of superkingdom-specific structures was low in each superkingdom (median *f*-values = 0.01–0.34), with minimum average *f*-values for Bacteria and maximum for Eukarya (Figure 3(a), A, B, and E). Remarkably, out of the 164 Bacteria-specific structures, none, but one, was present in >50% of the proteomes (Figure 3(a), B). The absence of an expected homogenous distribution strongly suggests that the role of HGT and other homogenizing processes may be quite limited in shaping the evolution of bacterial proteomes. Eukaryal-specific structures were distributed with higher *f*-values (Figure 3(a), E). The relatively low spread of superkingdom-specific structures suggests that these structures were acquired independently and after divergence from the last common ancestors of each superkingdom.

Inferences drawn from boxplots of the *function* dataset (Figure 3(b)) supported the general conclusions derived from the *structure* dataset. The ABE distributions had high *f*-values, with those of Archaea (median *f* = 0.24) being considerably lower than those of Bacteria (0.57) and Eukarya (0.57) (Figure 3(b), ABE). Bacterial and eukaryal distributions were remarkably balanced, providing additional support to their recent divergence from a mutual ancestor. The median *f*-value in Archaea was lowest and could be explained by either high genome reduction events [26] or biases in the number of GO annotations for archaeal genomes. GOs are more reliably and extensively curated for Bacteria and Eukarya, and this factor could reduce the number of overall detections in archaeal genomes. However, comparing distributions of the *function* and *structure* datasets show that supporting results were consistent and suggest a limited impact of this possible shortcoming. Here, ABE distributions followed the pattern observed for FSFs and were therefore considered reliable. None of the AB, AE, and BE taxonomic groups showed balanced distributions (Figure 3(b), AB, AE, and BE). The AB taxonomic group harbored 100 GOs (~3-fold greater than corresponding structures) that were distributed with low popularity (Figure 3(b), AB). In general, these functions were more abundant in Bacteria compared to Archaea and thus suggested that some molecular activities were laterally transferred from Bacteria to Archaea (confirmed below). The AE taxonomic group failed to strongly support AE distributions in the *structure* dataset. This group included only 11 GOs that were relatively more abundant in eukaryal proteomes (Figure 3(b), AE). Finally, the BE taxonomic group also supported the increased prevalence of BE functions in eukaryal genomes compared to bacterial genomes (0.39 median versus 0.03), indicating either horizontal trace effects or biases introduced by GO annotation schemes (Figure 3(b), BE). However, the numbers of traits of the BE group were considerably greater than those of either the AB or AE groups and included a significantly large number of functions that were relatively widespread (*f* > 0.8) (Figure 3(b), BE). This was in sharp contrast with patterns in either AB or AE taxonomic groups. The subset of highly represented BE functions is therefore the most likely trace of an ancient vertical signature that unifies Bacteria and Eukarya as sister-groups in the ToL. This trace is remarkably consistent with the patterns obtained in the *structure* dataset (Figures 2(a) and 3(a)).

Finally, the superkingdom-specific functions were again distributed with low *f*-values. Archaea had only one unique GO that was present in 40% of the archaeal genomes (Figure 3(b), A). In sharp contrast, there were 162 bacterial and 852 eukaryal-specific GOs. Bacterial functions again showed evidence of very limited spread in organisms (Figure 3(b), B) challenging claims of widespread bacterial HGT. In turn, eukaryal functions were moderately widespread (Figure 3(b), E). These results are in line with earlier inferences regarding late and independent acquisition of superkingdom-specific traits.

### 3.3. Identifying Patterns of Horizontal Flux

Boxplot distributions provided useful clues regarding the divergence patterns of superkingdoms. However, they did not allow us to quantify the extent of horizontal versus vertical inheritance. Therefore, we calculated a difference in the *f*-value for all traits in the AB, AE, and BE taxonomic groups. If the difference between *f*-values was >0.6, the presence of the trait in both superkingdoms was considered the result of a probable HGT event. This threshold was set arbitrarily to include only those traits that were considerably more abundant in one superkingdom but scarcely present in the other. For example, the “t-snare proteins” superfamily [SCOP id: a.47.2], which is abundantly found in yeast and mammalian cells and forms bridges to mediate intracellular trafficking [43], had an *f*-value of 0.996 in eukaryotes implying that it was ubiquitous. However, it was only present in one of the 652 bacterial proteomes examined (*f* = 0.001) (Table S1, Supplementary Materials available online at http://dx.doi.org/10.1155/2013/648746). This most likely is an example of structure gain via HGT that occurred in the direction from Eukarya to Bacteria. Using this criterion, only one structure (“tRNA-intron endonuclease N-terminal domain-like” [d.75.1]) was acquired horizontally in Eukarya from Archaea in the AE taxonomic group, while 6 were transferred from Eukarya to Archaea (Table S1). Similarly, only one FSF was laterally transferred to Bacteria from Archaea (“Sulfolobus fructose-1,6-bisphosphatase-like” [d.280.1]) while none were acquired in reciprocity. Finally, Bacteria likely transferred 35 structures to eukaryotes while gained 52 in return (Table S1). The rest 237 structures did not show significant deviations in terms of spread in these taxonomic groups and were possibly acquired vertically or gained independently in evolution.

In terms of *function*, none of the GO traits were likely transferred to Bacteria from Archaea. However, 9 GOs were transfer candidates from Bacteria to Archaea (Table S2). Perhaps the most interesting among these was the lateral acquisition of “penicillin binding molecular activity” [GO:0008658] that was universally present in Bacteria but also present in 11% of the archaeal proteomes (Table S2). Similarly, no molecular function was transferred to Eukarya from Archaea, while only one GO (“dolichyl-diphosphooligosaccharide-protein glycotransferase activity” [GO:0004579]) was gained. Finally, 4 molecular functions were likely transferred from Bacteria to Eukarya and 28 were gained in return (Table S2). Overall, the inferred impact of horizontal transfer processes appeared to be quite limited and did not seriously invalidate our inferences. Moreover, horizontal contributions from Archaea to either Bacteria or Eukarya were minimal, which is consistent with the minimal sharing of traits described above (Figures 2 and 3). In comparison, both Bacteria and Eukarya exhibited higher levels of vertical and horizontal inheritance of traits and indicated a much stronger evolutionary association, a conclusion intimated by likely ancient endosymbiotic events.

### 3.4. Identifying Ancestral Traits Using Abundance Counts

**Figure 4:**
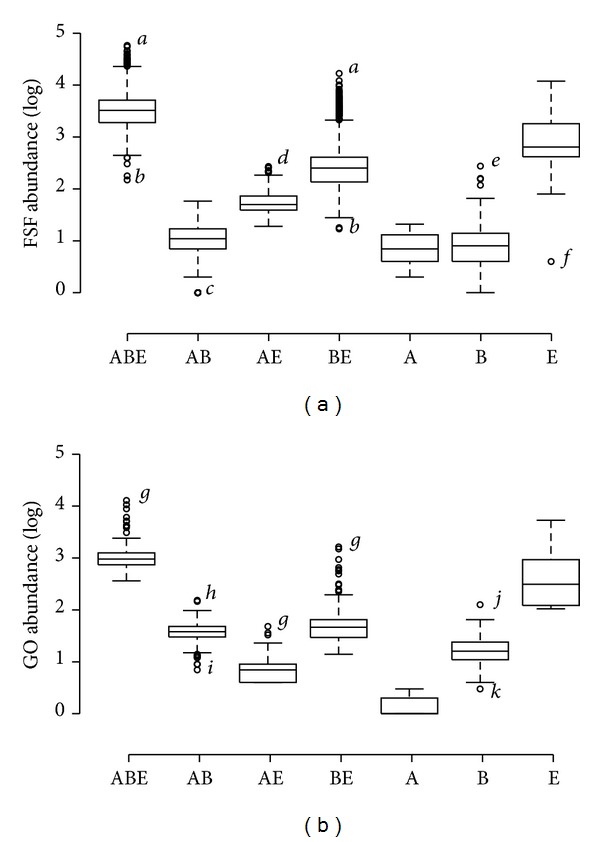
Boxplots comparing the log-transformed abundance values of structural (a) and functional (b) traits in the proteomes and functionomes of the seven Venn taxonomic groups. Italicized characters identify outliers with maximum and minimum abundance of traits in each group: *a, Takifugu rubripes; b, Cand. *Hodgkinia cicadicola Dsem; *c, Mycoplasma genitalium G37; d, Zea mays; e, Mycobacterium marinum; f, Guillardia theta; g, Homo sapiens; h, Rhodospirillum rubrum; i, Desulfurococcus kamchatkensis; j, Ralstonia eutropha; k, Thermosipho africanus.*

Traits that are of ancient origin are expected to be present in greater abundance than those acquired recently. This is true because traits appearing earlier have more time to accumulate in genomes and to increase their representation [6]. Thus, high abundance of traits in a particular Venn taxonomic group is indicative of the presence of relatively more ancient traits and an ancient origin. Therefore, genomic abundance can be used as one proxy to estimate the age of taxonomic groups. We calculated the abundance of traits present in each proteome and functionome and represented these values in boxplot distributions (Figure 4). The median abundance value was highest for the ABE taxonomic group in both the *structure* (Figure 4(a)) and *function* (Figure 4(b)) datasets, again supporting that this group retains most of the urancestral traits that have relished maximum time to multiply and become abundant in modern proteomes and functionomes. The BE group always harbored traits in much greater abundance compared to the AB and AE groups (Figure 4). Finally, Eukarya-specific traits were significantly enriched in the eukaryal proteomes and functionomes and were detected in much greater abundance compared to the genomic abundance of either Archaea-specific or Bacteria-specific traits (Figure 4). This result confirms the existence of a strong vertical trace in modern cells in the direction from ABE to BE and to E. It is likely that eukaryotes retained the majority of the most ancient traits that were progressively lost in akaryal organisms, beginning in Archaea and manifesting much later in Bacteria. Previous phylogenomic analyses have confirmed strong reductive trends in the akaryal proteomes [26, 28, 35]. Evolution of Archaea has also been linked to genome reduction events that started very early in evolution and before the appearance of the BE taxonomic group [28, 35]. However, the relatively late loss of traits in Bacteria is intriguing. Several bacterial species are known to have adapted a parasitic lifestyle following genome reduction [44]. Thus, gene loss in Bacteria is likely an ongoing evolutionary process hinting towards a major secondary evolutionary transition. This was also manifested in the very poor spread of Bacteria-specific traits (Figure 3).

We provide evidence for late loss in Bacteria by closely examining the AE traits. The majority of the 38 AE FSFs and 11 GOs are enriched in informational functions (e.g., translation initiation, ribosomal proteins, DNA binding proteins, and proteins involved in DNA replication; Tables S3 and S4). This result is consistent with existing knowledge. Indeed, Archaea and Eukarya are more related to each other in terms of informational processes, while Bacteria and Eukarya resemble each other metabolically [45]. Thus, the high popularity of AE FSFs could be due to biases attributed to late differential loss of structures in these functional categories. For example, the 11 AE GOs include crucial molecular functions such as “DNA polymerase processivity factor activity [GO:0030337]” and “tRNA-intron endonuclease activity [GO:0000213].” The former is a regulator of the replication fork [46, 47] while the latter is involved in processing tRNA introns [48]. Both of these activities could be linked to late losses in Bacteria, as they seem centrally important functions in cells. Therefore, while HGT, convergent evolution and coevolution of BE traits seems less likely, we cannot rule out the possibility of extensive genome reduction in akaryal species.

### 3.5. Tracking the Vertical Trace

**Figure 5:**
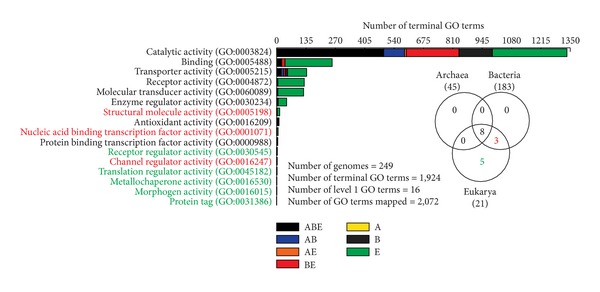
Bar plots illustrating the breakdown of terminal GOs in the seven taxonomic groups for level 1 GO terms. A total of 1,871 out of 1,924 GOs (97.24%) could be reliably mapped to their parents. Level 1 GOs that could not be mapped include “D-alanyl carrier activity [GO:0036370],” “electron carrier activity [GO:0009055],” “chemoattractant activity [GO:0042056],” “chemorepellent activity [GO:0045499],” and “nutrient reservoir activity [GO:0045735].” Note that terminal GOs may have more than one parent. The Venn diagram shows that none of the A, B, AB, and AE taxonomic groups uniquely code for any level 1 GO term.

To further dissect the evolution of Venn taxonomic groups, we mapped the 1,924 terminal GOs to 16 level 1 parent GO terms.  Figure 5 shows the distribution of terminal GOs, indexed by taxonomic group, in each of the 16 parent categories. This exercise confirmed the inferences drawn from earlier experiments and highlighted the direction of the vertical trace. Remarkably, only ABE, BE, and E were enriched in level 1 molecular functions while the majority of the terminal GO terms could be identified as either “catalytic activity [GO:0003824]” or “binding [GO:0005488]” (Figure 5). This is an interesting result. A previous analysis by Kim and Caetano-Anollés [25] confirmed that these two molecular activities appeared first in evolution and were shared by all organisms. In comparison, the more derived molecular activities first appeared in the BE taxonomic group (e.g., “structural molecule activity [GO:0005198],” “nucleic acid binding transcription factor activity [GO:0001071],” and “channel regulator activity [GO:0016247]),” while the recent innovations occurred uniquely in Eukarya (e.g., “receptor regulator activity [GO:0030545], “translation regulator activity [GO:0045182],” “metallochaperone activity [GO:0016530],” “morphogen activity [GO:0016015],” and “protein tag [GO:0031386]”). In contrast, none of the AB, AE, A, and B taxonomic groups uniquely harbored a level 1 molecular function (Figure 5). Remarkably, a significant proportion of the BE terminal GOs was devoted to the most ancient catalytic and binding activities (Figure S1). In comparison, “transporter activity [GO:0005215]” was found to be over-represented in the AB group while AE was numerically much smaller (Figure S1). These findings confirm the existence of a vertical trace from ABE to BE and finally to E (also supported by the *structure* dataset). Akaryal ancestors likely diverged from this trace by following paths towards genome reductions while eukaryotes enriched their repertoires by engaging in gene duplication events and exploring novel domain combinations [12, 49].

### 3.6. Validating Inferences with Evolutionary Timelines

**Figure 6:**
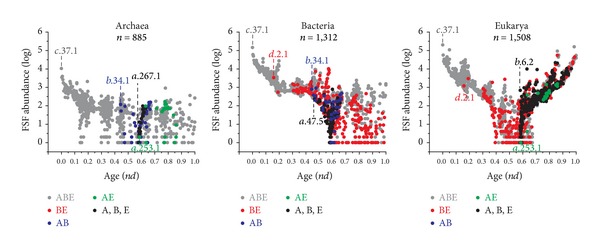
Evolutionary timelines highlighting the abundance of FSFs in superkingdom taxonomic groups. Evolutionary age (*nd*) was calculated from a phylogenetic tree of protein domains describing the evolution of 1,733 FSFs (taxa) in 981 organisms (characters) (see [26, 28, 35] for technical details). SCOP alphanumeric identifiers were used to identify the most ancient FSF in each taxonomic group. In case of multiple FSFs of same age, only the FSF with maximum abundance was labeled. *c.37.1* is the P-loop containing NTP hydrolase FSF; *b.34.1* is the C-terminal domain of transcriptional repressors FSF; *a.267.1* is the topoisomerase V catalytic domain-like FSF; *a.253.1* is the AF0941-like FSF; *d.2.1* is the Lysozyme-like FSF; *a.47.5* is the FlgN-like FSF; *b.6.2* is the major surface antigen p30, SAG1.

To validate our ahistorical comparative approach, we unfolded the appearance of FSF and GO traits in evolutionary time (*nd*), while plotting their genomic abundance in each superkingdom. The historical analyses of FSF evolution (Figure 6) and GO terminal terms (data not shown) were congruent and revealed two clear patterns: (1) a pattern of ancient genomic loss embodying the early rise of the BE taxonomic group (red circles), which generally involved traits with abundance levels that were at least an order of magnitude higher than the levels of other taxonomic groups (e.g., AE and AB); and (2) a canonical pattern of appearance of superkingdom-specific traits that revealed the rise of early bacterial novelties followed by the joint appearance of unique novelties in Archaea and Eukarya. This historical analysis therefore supports the ancient vertical trace identified by comparative analysis that flows from the ABE group to the BE and E groups. These three groups were distributed with maximum abundance values in timelines indicating retention of large number of traits from the common ancestor. This vertical trace defines an ancient stem line of descent responsible for the early origination of archaeal lineages and bacterial novelties, which reconciles the canonical and archaeal rooting of the ToL. The ahistorical analysis however was unable to predict the canonical pattern, since the comparative analysis of trait distribution in Venn taxonomic groups, superkingdoms, and organisms cannot accommodate competing hypotheses of rooting that manifest at different times in evolution. The plots of Figure 6 also revealed a marked increase in the abundance of FSFs late in eukaryal evolution, which can be explained by the remarkable development of multidomain protein structures and their associated functions [12, 49]. The combinatorics of domains and functions are the likely culprit of the biphasic patterns we observed when we focused on Eukarya.

## 4. Discussion

Our approach is simple (Figure 1). It does not involve computation of a sequence alignment or use of complex data matrices for phylogenetic reconstruction. Instead, it focuses on the census of molecular (structural and functional) traits in the genomes of modern cells. The fundamental principle of analysis is the use of trait distributions in Venn taxonomic groups to explain vertical evolutionary traces, the use of *f*-values to explain horizontal traces, and the use of trait abundance as a proxy for age. The sequential combination of these approaches dissects the most likely scenario of diversification of superkingdoms, without invoking a phylogenetic framework of analysis.

Our comparative genomic exercise shows evidence in favor of a common ancestry for cells and establishes the deep branching patterns of the ToL. The genetic complexity of Bacteria and Eukarya hints towards a strong and ancient evolutionary association between the two superkingdoms. This association is stronger than the associations of other superkingdoms. Our findings are also compatible with an evolutionary scenario in which Archaea emerged as the first superkingdom of life by diverging from a primordial stem line of descent that originated in the urancestor [26, 28]. This line likely encountered extreme temperatures that affected its proteomic growth, hampering the acquisition of new molecular traits in those environments. Under such hostile conditions, the persistence strategy of the emerging archaeal cells was most likely survival rather than enrichment [50]. This explains why we observed the lowest number of traits in extant archaeal species. In contrast, both Bacteria and Eukarya shared a protracted coevolutionary history. Their diversification occurred well after the primordial split of Archaea from the urancestral line. Bacteria followed a path towards exploring a diverse range of habitats, which enabled high rates of gene discovery. This explains the high numbers of unique bacterial traits that are unequally distributed among bacterial species. Bacterial species also engaged in genome reductive processes and simplified their trait representations. This probably occurred well after their divergence from the primordial stem line. Finally, eukaryotes evolved by (i) increasing the abundance of ancient traits (via gene duplications and domain rearrangements), (ii) discovering novel traits, or (iii) both. These findings falsify an evolutionary scenario of first appearance of bacterial cells [2, 3] or the fusion hypotheses linked to the origin of eukaryotes (e.g., [33]), as none seems compatible with our data. However, we did not consider the roles that viruses may have played during cellular evolution. Viruses are known to contribute to the genetic diversity of cells and are believed to be very ancient [35, 51–53]. We will accomplish this task in the near future.

Genome reduction is an ongoing evolutionary process that often triggers lifestyle transitions in cells (e.g., from free-living to intracellular parasites [44]). We propose that genome streamlining played a key role in the evolution of akaryotes, especially Archaea. Our data show that the BE taxonomic group was enriched in molecular traits compared to the relatively poor representations of FSFs and GOs in the AB and AE groups (Figure 2). In fact, evolutionary timelines revealed that the BE group appeared very early in evolution and was correlated with high abundance levels of BE FSFs in the bacterial and eukaryal proteomes (Figure 6). These findings were taken as an indication of loss of traits in Archaea that occurred very early in evolution. While it can be argued that such losses could have occurred much later in archaeal lineages and after their diversification from Bacteria, our comparative and evolutionary data indicate that this may not be very likely. The loss of ancient traits late in evolution is phylogenetically costly as it implies loss of many genes and proteins that have accumulated during the course of evolution to perform a particular molecular task. In comparison, loss of ancient traits early in evolution is more parsimonious and complies with the principle of spatiotemporal continuity. An alternative explanation, however, could be the confounding effects of HGT processes. However, it was shown recently that a large number of ribosomal proteins were unevenly distributed in archaeal species [54, 55]. Because ribosomal proteins are generally refractory to HGT, their patchy and uneven distribution in archaeal lineages is better explained by differential loss from a more complex archaeal ancestor. Taken together, these findings strongly suggest that primordial reductive evolutionary processes have tailored archaeal evolution.

When placed along evolutionary timelines of trait innovation (Figure 6), Venn taxonomic groups uncovered a remarkable pattern that could not be dissected with the comparative genomic approach. This hidden pattern embodies the primordial rise of Bacteria-specific traits followed much later by the concurrent appearance of Archaea-specific and Eukarya-specific innovations. This important succession supports the “canonical” rotting of the ToL in which Bacteria occupy the most basal positions while Archaea and Eukarya emerge as derived sister-groups [2, 3]. From a cladistics perspective, traits unique to a superkingdom are autapomorphies, derived features that are unique to terminal groups. These autapomorphies cannot be used to reconstruct trees in phylogenetic analysis or dissect the alternative evolutionary scenarios of our comparative genomic approach. In comparison, FSFs and GOs that are shared by any two superkingdoms reflect synapomorphies (shared and derived features) that allow both historical (phylogenetic) and ahistorical (comparative) inferences. We note that traits uniquely shared by any two superkingdoms can arise either by the gain of the feature in two superkingdoms or by the loss in one. Abundance levels and *f*-distribution patterns support the latter scenario, especially if the loss involves an ancient trait. Thus, an early primordial loss of FSFs and GO synapomorphies in Archaea embeds later on the early gain of autapomorphies in Bacteria.

The hidden canonical pattern of Figure 6 was already reported in an exhaustive structural phylogenomic exploration of domain evolution at fold and FSF levels of structural abstraction [26], which prompted the definition of three epochs in the evolution of proteins and the organismal world and a number of hypotheses of origin. In the first* “architectural diversification” *epoch, the emerging organismal community accumulated a rich toolkit of protein structures and functions. This communal world resembled the ancient world of multiphenotypical precells proposed by Kandler [56] that inspired Woese's more advanced scenarios of early cellular evolution [57]. However, and in contrast with the simple cellular systems sought by Kandler and Woese, the precell molecular make up that was inferred from our phylogenomic analysis was extremely rich in complex structures and functions [29]. This richness is expressed today in the sizable number of structures and functions that are shared by all superkingdoms and are revealed by our comparative exploration. Towards the end of the architectural diversification epoch, the pervasive loss of domain structures in subgroups of the urancestral precell population resulted in primordial archaeal grades, groups of diversifying organisms in active transition that were at first unified by the physiological complexity of the urancestral community but later on gained the cellular cohesiveness needed to establish lineages and true patterns of organismal diversification. While it may prove difficult to establish the time when these “thresholds” (sensu [57]) were crossed by the primordial archaeal grades as these were stemming from the urancestral stem line, the early process of reductive evolution left deep historical signatures in the make up of the archaeal organisms that are embedded in the timelines of domain structures [26]. The second* “superkingdom specification” *epoch brought the first Bacteria-specific domain structures and later on the concurrent appearance of Archaea-specific and Eukarya-specific structures. This canonical pattern of appearance of superkingdom-specific structures, which unfolded in the absence of early and major reductive evolutionary tendencies, signals a time in which the emerging superkingdoms were being molded by innovation. During this epoch, grades turned into clades and the precell “swap shop” strategy was gradually replaced by organismal cohesiveness. Marked decreases in *f*-values during this time suggested that lineage sorting occurred more frequently in the growing number of lineages. Finally, in the* “organismal diversification”* epoch, commitment to strategies and lifestyles enhanced even further the divide between superkingdoms and weakened the contribution of the stem line of descent. Two forces of particular significance play crucial roles during this final epoch, the combinatorial use of domains as modules in multidomain proteins of Eukarya [12, 49] that is responsible for the high abundance levels and the biphasic patterns of Figure 6 and the HGT-driven combinatorial exchange of protein repertoires in lineages of Bacteria [26] that minimizes trait distribution in Figure 3.

We end by emphasizing that our comparative genomic inferences have been ratified previously by phylogenetic tree reconstructions (e.g., [11–13, 17, 22, 26, 28]) and thus establish the power of our methodology. However, our analysis depends upon the accuracy and sampling of structures and functions and the reliability of the datasets. The *function* dataset, in particular, is dependent upon the stability of GO annotations and is biased towards eukaryal organisms that are more carefully annotated. To minimize this factor, we sampled 183 bacterial and 45 archaeal functionomes in comparison to only 21 eukaryotes. Despite the huge number of akaryal functionomes in our dataset, we were still able to highlight the incredible enrichment of eukaryal repertoires. Moreover, inferences drawn from *function* were in agreement with *structure* and both should be considered reliable.

While tracing back evolutionary history from the present to the first cell is a complex problem, inferring the patterns of species diversification by comparing the use and reuse of molecular traits in extant cells must be considered a robust inferencial approach that is free from many of the external assumptions and technical problems faced when reconstructing phylogenetic trees. The only shortcoming may be one of interpretation, which we here showcase with the scenarios of origin we have discussed. However, we have tried to restrict our statements to scenarios that seem most compatible with the given data. An example is using a threshold of 60% difference in the popularity of traits to detect HGT-derived structures and functions. This criterion was set arbitrarily to identify only the most likely HGT-transfers but may have resulted in failure to detect some of the true HGT-acquired traits, especially for those where both intersuperkingdom and intrasuperkingdom transfers occurred rapidly. Although such events are less likely, they may still be occurring. However, detection of such transfers is a hard problem and cannot be reliably confirmed without experimental evidence. Given the conservation levels of structural and functional traits and the relatively poor repertoire of likely HGT-acquired features (Tables S1 and S2), we safely assume that this factor did not seriously compromise our inferences. Finally, our approach is a systematic application of morphological analyses that were initially used to classify higher-order organisms. Future work should be focused on advanced applications of our approach for reaching a consensus regarding the evolution of cells.

## 5. Conclusions

We inferred evolutionary patterns by examining the spread of molecular features in contemporary organisms. The analysis revealed a common origin for all cells, the early divergence of Archaea, and a sister relationship between Bacteria and Eukarya. Archaeal evolution was primarily influenced by genome reduction while that of Bacteria by two contrasting phases: (i) a period of early innovation that coincides with the rise and diversification of the bacterial superkingdom, and (ii) a postdivergence period of this lineage exhibiting relatively late genome reduction events. The branch leading to modern eukaryotes was minimally affected by reductive pressure and retained the majority of the ancestral traits. Eukaryotes further enriched the genomic abundance of these traits by engaging in gene duplication and domain rearrangement processes and by discovering novel structures and molecular activities. Traces of all of these events could be reliably detected in modern proteomes and functionomes. In particular, a strong vertical trace from the urancestor to the stem line unifying Bacteria and Eukarya and the ancestor of Eukarya could be inferred. This strong vertical trace strongly supports the existence of a stem line of descent, from which all three superkingdoms emerged, very much in line with Kandler's ideas of an aboriginal precellular line of early biochemical evolution that was undergoing cellularization [56]. Finally, nonvertical evolutionary processes seemed to have played only limited roles during defining steps of cellular evolution. The comparative framework enables exploration of deep evolutionary histories without invoking tree reconstruction algorithms and external hypotheses of evolution. This approach is in line with various published phylogenetic analyses and provides strong support to theories favoring an archaeal origin of diversified life.

## Supplementary Material

TABLE S1: List of FSFs that were likely acquired via HGT in the AB, AE, and BE taxonomic groups. Both the SCOP and alphanumeric identifiers were used to define FSFs. HGT direction was inferred from *f_difference_*, which is the difference in *f-*values between *f_former_*(e.g. A in the AB taxonomic group) and flatter (B in AB).
TABLE S2: List of GOs that were likely acquired via HGT in the AB, AE, and BE taxonomic groups. HGT direction was inferred from *f_difference_*, which is the difference in *f*values between *f_former_* (e.g. A in the AB taxonomic group) and *f_latter_* (B in AB).
TABLE S3: List of FSFs that were uniquely detected in the proteomes of the AE taxonomic group.
TABLE S4: List of terminal GOs that were uniquely detected in the functionomes of the AE taxonomic group.
FIGURE S1: Pie charts displaying the distribution of terminal GOs in level-1 parent terms. Numbers indicate total number of terminal GOs annotated to each parent term category. Terms may be mapped to more than one parent.

## Figures and Tables

**Table 1:** List of universal FSFs that were present in all proteomes of the *structure* dataset.

No.	SCOP Id	FSF Id	FSF description
1	52540	c.37.1	P-loop containing nucleoside triphosphate hydrolases
2	50249	b.40.4	Nucleic acid-binding proteins
3	53067	c.55.1	Actin-like ATPase domain
4	51905	c.3.1	FAD/NAD(P)-binding domain
5	53098	c.55.3	Ribonuclease H-like
6	54211	d.14.1	Ribosomal protein S5 domain 2-like
7	55681	d.104.1	Class II aaRS and biotin synthetases
8	50447	b.43.3	Translation proteins
9	54980	d.58.11	EF-G C-terminal domain-like
10	50104	b.34.5	Translation proteins SH3-like domain
11	50465	b.44.1	EF-Tu/eEF-1alpha/eIF2-gamma C-terminal domain
12	55174	d.66.1	Alpha-L RNA-binding motif
13	54768	d.50.1	dsRNA-binding domain-like
14	55257	d.74.3	RBP11-like subunits of RNA polymerase
15	52080	c.12.1	Ribosomal proteins L15p and L18e
16	54686	d.41.4	Ribosomal protein L16p/L10e
17	54843	d.55.1	Ribosomal protein L22

**Table 2:** List of universal GOs that were present in all functionomes of the *function* dataset.

No.	GO Id	GO description
1	GO:0005524	ATP binding
2	GO:0008270	zinc ion binding
3	GO:0000287	magnesium ion binding
4	GO:0005525	GTP binding
5	GO:0004222	metalloendopeptidase activity
6	GO:0010181	FMN binding
7	GO:0030145	manganese ion binding
8	GO:0003924	GTPase activity
9	GO:0003887	DNA-directed DNA polymerase activity
10	GO:0004252	serine-type endopeptidase activity
11	GO:0003746	translation elongation factor activity
12	GO:0009982	pseudouridine synthase activity
13	GO:0004523	ribonuclease H activity
14	GO:0004826	phenylalanine-tRNA ligase activity
15	GO:0004821	histidine-tRNA ligase activity
16	GO:0004820	glycine-tRNA ligase activity
17	GO:0004824	lysine-tRNA ligase activity
18	GO:0004831	tyrosine-tRNA ligase activity
19	GO:0004618	phosphoglycerate kinase activity
20	GO:0004634	phosphopyruvate hydratase activity
21	GO:0004749	ribose phosphate diphosphokinase activity
22	GO:0003952	NAD+ synthase (glutamine-hydrolyzing) activity
23	GO:0004815	aspartate-tRNA ligase activity
24	GO:0004807	triose-phosphate isomerase activity
25	GO:0004813	alanine-tRNA ligase activity
26	GO:0003917	DNA topoisomerase type I activity
