# Supplementary material for: Comparative Analysis of Proteomes and Functionomes Provides Insights into Origins of Cellular Diversification
Source: Archaea. 2013 Dec 31;2013:648746. doi: 10.1155/2013/648746 (PMC3892558; doi:10.1155/2013/648746)
Supplement: Supplementary file 1 — TABLE S1: List of FSFs that were likely acquired via HGT in the AB, AE, and BE taxonomic groups. Both the SCOP and alphanumeric identifiers were used to define FSFs. HGT direction was inferred from fdifference, which is the difference in f-values between fformer(e.g. A in the AB taxonomic group) and flatter (B in AB). TABLE S2: List of GOs that were likely acquired via HGT in the AB, AE, and BE taxonomic groups. HGT direction was inferred from fdifference, which is the difference in fvalues between fformer (e.g. A in the AB taxonomic group) and flatter (B in AB). TABLE S3: List of FSFs that were uniquely detected in the proteomes of the AE taxonomic group. TABLE S4: List of terminal GOs that were uniquely detected in the functionomes of the AE taxonomic group. FIGURE S1: Pie charts displaying the distribution of terminal GOs in level-1 parent terms. Numbers indicate total number of terminal GOs annotated to each parent term category. Terms may be mapped to more than one parent. [file 648746.f1.pdf]

Supplementary Material

TABLE S1: List of FSFs that were likely acquired via HGT in the AB, AE, and BE taxonomic groups. Both the SCOP and alphanumeric identifiers were used to define FSFs. HGT direction was inferred from $f_{difference}$, which is the difference in f-values between f_{former} (e.g. A in the AB taxonomic group) and f_{latter} (B in AB).

Taxonomic group	SCOP Id	FSF Id	FSF name	f_{former}	f_{latter}	$f_{difference}$	HGT direction
AB	111249	d.280.1	Sulfolobus fructose-1,6-bisphosphatase-like	0.7143	0.0353	0.679	A to B
AE	55267	d.75.1	tRNA-intron endonuclease N-terminal domain-like	0.9429	0.0154	0.9274	A to E
AE	118310	g.80.1	AN1-like Zinc finger	0.3143	0.9498	-0.6355	E to A
AE	47157	a.23.4	Mitochondrial import receptor subunit Tom20	0.0143	0.7066	-0.6923	E to A
AE	55418	d.86.1	eIF4e-like	0.1286	1	-0.8714	E to A
AE	101576	b.132.1	Supernatant protein factor (SPF), C-terminal domain	0.0143	0.8996	-0.8853	E to A
AE	56741	e.15.1	Eukaryotic DNA topoisomerase I, N-terminal DNA-binding fragment	0.0143	0.9498	-0.9355	E to A
AE	109993	a.222.1	VPS9 domain	0.0143	0.9846	-0.9703	E to A
BE	69705	d.202.1	Transcription factor NusA, N-terminal domain	0.9954	0.0039	0.9915	B to E
BE	48295	a.4.12	TrpR-like	0.9831	0.0039	0.9793	B to E
BE	74982	b.111.1	Small protein B (SmpB)	0.9985	0.0347	0.9637	B to E
BE	48024	a.81.1	N-terminal domain of DnaB helicase	0.9877	0.027	0.9607	B to E
BE	47789	a.60.3	C-terminal domain of RNA polymerase alpha subunit	0.9954	0.0502	0.9452	B to E
BE	46557	a.2.1	GreA transcript cleavage protein, N-terminal domain	0.9555	0.0116	0.9439	B to E
BE	111304	e.49.1	Recombination protein RecR	0.9509	0.0116	0.9393	B to E
BE	56519	d.175.1	Penicillin binding protein dimerisation domain	0.9463	0.027	0.9193	B to E
BE	54752	d.48.1	RecA protein, C-terminal domain	0.9847	0.0734	0.9113	B to E
BE	82679	d.58.42	N-utilization substance G protein NusG, N-terminal	0.9954	0.0965	0.8989	B to E

			domain				
BE	46992	a.7.6	Ribosomal protein S20	0.9831	0.0965	0.8866	B to E
BE	143517	d.315.1	TRCF domain-like	0.9325	0.0618	0.8707	B to E
BE	102735	d.241.2	Trigger factor ribosome-binding domain	0.9862	0.1158	0.8704	B to E
BE	63418	c.98.1	MurE/MurF N-terminal domain	0.9371	0.0734	0.8638	B to E
BE	141259	b.34.18	CarD-like	0.9371	0.0734	0.8638	B to E
BE	88946	a.177.1	Sigma2 domain of RNA polymerase sigma factors	0.9985	0.1429	0.8556	B to E
BE	81886	a.172.1	Helical scaffold and wing domains of SecA	0.9939	0.139	0.8549	B to E
BE	75420	d.52.4	YhbC-like, N-terminal domain	0.8834	0.0347	0.8487	B to E
BE	55653	d.99.1	Ribosomal protein L9 C-domain	0.9939	0.1506	0.8433	B to E
BE	81767	a.162.1	Pre-protein crosslinking domain of SecA	0.9939	0.1506	0.8433	B to E
BE	109998	a.223.1	Triger factor/SurA peptide-binding domain-like	0.9954	0.166	0.8294	B to E
BE	82607	d.222.1	YbaB-like	0.9126	0.1274	0.7852	B to E
BE	68912	a.140.3	Rho N-terminal domain-like	0.8436	0.0618	0.7818	B to E
BE	103088	d.79.7	OmpA-like	0.7945	0.0425	0.752	B to E
BE	74942	b.38.2	YhbC-like, C-terminal domain	0.7515	0.0116	0.74	B to E
BE	141523	b.160.1	L,D-transpeptidase catalytic domain-like	0.7362	0.0154	0.7208	B to E
BE	82693	d.58.44	Multidrug efflux transporter AcrB pore domain; PN1, PN2, PC1 and PC2 subdomains	0.7454	0.0463	0.6991	B to E
BE	82714	d.225.1	Multidrug efflux transporter AcrB TolC docking domain; DN and DC subdomains	0.7285	0.0463	0.6822	B to E
BE	111369	f.46.1	HlyD-like secretion proteins	0.7163	0.0386	0.6776	B to E
BE	109635	a.2.14	DnaK suppressor protein DksA, alpha-hairpin domain	0.681	0.0039	0.6771	B to E
BE	54523	d.24.1	Pili subunits	0.6794	0.0232	0.6563	B to E
BE	69765	d.79.5	IpsF-like	0.8282	0.1815	0.6468	B to E
BE	141868	c.1.33	EAL domain-like	0.6764	0.0347	0.6416	B to E
BE	69055	a.69.3	1-deoxy-D-	0.8037	0.1699	0.6338	B to E

			xylulose-5-phosphate reductoisomerase, C-terminal domain				
BE	110399	c.1.31	ThiG-like	0.6856	0.0656	0.6199	B to E
BE	55895	d.124.1	Ribonuclease Rh-like	0.1871	0.7992	-0.6121	E to B
BE	55387	d.82.2	Frataxin/Nqo15-like	0.2408	0.8533	-0.6125	E to B
BE	142921	d.297.1	WGR domain-like	0.0767	0.6988	-0.6222	E to B
BE	140959	a.266.1	Indolic compounds 2,3-dioxygenase-like	0.1933	0.8185	-0.6253	E to B
BE	48056	a.86.1	Di-copper centre-containing domain	0.0552	0.6911	-0.6359	E to B
BE	69848	d.209.1	LCCL domain	0.0046	0.6448	-0.6402	E to B
BE	57552	g.20.1	Blood coagulation inhibitor (disintegrin)	0.0031	0.668	-0.6649	E to B
BE	55724	d.107.1	Mog1p/PsbP-like	0.089	0.7568	-0.6678	E to B
BE	110836	d.267.1	Hypothetical protein SAV1430	0.2209	0.8919	-0.671	E to B
BE	63862	b.82.6	Thiamin pyrophosphokinase, substrate-binding domain	0.1779	0.8571	-0.6792	E to B
BE	50405	b.42.5	Actin-crosslinking proteins	0.0552	0.7375	-0.6822	E to B
BE	55550	d.93.1	SH2 domain	0.0015	0.6873	-0.6857	E to B
BE	47672	a.48.2	Transferrin receptor-like dimerisation domain	0.0184	0.7104	-0.692	E to B
BE	47874	a.65.1	Annexin	0.0046	0.7027	-0.6981	E to B
BE	109885	a.216.1	I/LWEQ domain	0.0015	0.722	-0.7205	E to B
BE	49354	b.1.11	PapD-like	0.2132	0.9537	-0.7405	E to B
BE	48366	a.117.1	Ras GEF	0.0046	0.8031	-0.7985	E to B
BE	81496	f.23.11	Cytochrome c1 subunit of cytochrome bc1 complex (Ubiquinol-cytochrome c reductase), transmembrane anchor	0.0276	0.8263	-0.7986	E to B
BE	48065	a.87.1	DBL homology domain (DH-domain)	0.0015	0.8069	-0.8054	E to B
BE	54447	d.18.1	ssDNA-binding transcriptional regulator domain	0.0307	0.8571	-0.8265	E to B
BE	63491	a.7.7	BAG domain	0.0015	0.8571	-0.8556	E to B
BE	47027	a.11.1	Acyl-CoA binding protein	0.0859	0.9653	-0.8794	E to B
BE	47592	a.42.1	SWIB/MDM2	0.0644	0.9498	-0.8854	E to B

			domain				
BE	47762	a.59.1	PAH2 domain	0.0015	0.888	-0.8865	E to B
BE	48225	a.102.2	Seven-hairpin glycosidases	0.0092	0.9073	-0.8981	E to B
BE	53036	c.52.3	Eukaryotic RPB5 N-terminal domain	0.0031	0.9189	-0.9159	E to B
BE	55277	d.76.1	GYF domain	0.0138	0.9498	-0.936	E to B
BE	55637	d.97.1	Cell cycle regulatory proteins	0.0015	0.9382	-0.9367	E to B
BE	90209	g.41.11	Ran binding protein zinc finger-like	0.0015	0.9382	-0.9367	E to B
BE	117289	b.69.14	Nucleoporin domain	0.0215	0.9691	-0.9476	E to B
BE	81383	a.158.1	F-box domain	0.0092	0.9614	-0.9522	E to B
BE	69000	a.24.15	FAD-dependent thiol oxidase	0.0015	0.9575	-0.956	E to B
BE	82109	b.42.6	MIR domain	0.0031	0.9614	-0.9583	E to B
BE	63748	b.34.9	Tudor/PWWP/MBT	0.0061	0.9653	-0.9591	E to B
BE	74924	b.34.10	Cap-Gly domain	0.0015	0.9653	-0.9637	E to B
BE	56019	d.135.1	The spindle assembly checkpoint protein mad2	0.0077	0.973	-0.9653	E to B
BE	47769	a.60.1	SAM/Pointed domain	0.0092	0.9768	-0.9676	E to B
BE	48350	a.116.1	GTPase activation domain, GAP	0.0046	0.9846	-0.98	E to B
BE	57850	g.44.1	RING/U-box	0.0199	1	-0.9801	E to B
BE	101447	a.207.1	Formin homology 2 domain (FH2 domain)	0.0061	0.9884	-0.9823	E to B
BE	54236	d.15.1	Ubiquitin-like	0.0138	0.9961	-0.9823	E to B
BE	48425	a.118.3	Sec7 domain	0.0107	0.9961	-0.9854	E to B
BE	54695	d.42.1	POZ domain	0.0015	0.9884	-0.9869	E to B
BE	48464	a.118.9	ENTH/VHS domain	0.0015	0.9884	-0.9869	E to B
BE	49348	b.1.10	Clathrin adaptor appendage domain	0.0046	0.9923	-0.9877	E to B
BE	54495	d.20.1	UBC-like	0.0107	1	-0.9893	E to B
BE	49562	b.7.1	C2 domain (Calcium/lipid-binding domain, CaLB)	0.0061	0.9961	-0.99	E to B
BE	81995	b.2.8	beta-sandwich domain of Sec23/24	0.0015	0.9923	-0.9907	E to B
BE	82919	g.41.10	Zn-finger domain of Sec23/24	0.0015	0.9923	-0.9907	E to B
BE	74788	a.118.17	Cullin repeat-like	0.0015	0.9923	-0.9907	E to B
BE	47661	a.47.2	t-snare proteins	0.0015	0.9961	-0.9946	E to B
BE	50729	b.55.1	PH domain-like	0.0015	0.9961	-0.9946	E to B

TABLE S2: List of GOs that were likely acquired via HGT in the AB, AE, and BE taxonomic groups. HGT direction was inferred from $f_{difference}$, which is the difference in f-values between f_{former} (e.g. A in the AB taxonomic group) and f_{latter} (B in AB).

Taxonomic group	GO Id	GO name	f_{former}	f_{latter}	$f_{difference}$	HGT direction
AB	GO:0009381	excinuclease ABC activity	0.3778	1	-0.6222	B to A
AB	GO:0008855	exodeoxyribonuclease VII activity	0.2444	0.9235	-0.6791	B to A
AB	GO:0019134	glucosamine-1-phosphate N-acetyltransferase activity	0.1556	0.8634	-0.7078	B to A
AB	GO:0008965	phosphoenolpyruvate-protein phosphotransferase activity	0.0444	0.765	-0.7206	B to A
AB	GO:0008760	UDP-N-acetylglucosamine 1-carboxyvinyltransferase activity	0.0222	0.8306	-0.8084	B to A
AB	GO:0008881	glutamate racemase activity	0.0222	0.8852	-0.863	B to A
AB	GO:0008658	penicillin binding	0.1111	1	-0.8889	B to A
AB	GO:0008784	alanine racemase activity	0.0444	0.9836	-0.9392	B to A
AB	GO:0008763	UDP-N-acetylmuramate-L-alanine ligase activity	0.0222	0.9945	-0.9723	B to A
AE	GO:0004579	dolichyl-diphosphooligosaccharide-protein glycotransferase activity	0.1111	1	-0.8889	E to A
BE	GO:0008685	2-C-methyl-D-erythritol 2,4-cyclodiphosphate synthase activity	0.9071	0.0952	0.8119	B to E
BE	GO:0030604	1-deoxy-D-xylulose-5-phosphate reductoisomerase activity	0.9016	0.0952	0.8064	B to E
BE	GO:0003688	DNA replication origin binding	1	0.2857	0.7143	B to E
BE	GO:0003919	FMN adenylyltransferase activity	1	0.3333	0.6667	B to E
BE	GO:0004115	3',5'-cyclic-AMP phosphodiesterase activity	0.0984	0.7143	-0.6159	E to B
BE	GO:0018738	S-formylglutathione hydrolase activity	0.3333	0.9524	-0.619	E to B
BE	GO:0031418	L-ascorbic acid binding	0.3716	1	-0.6284	E to B
BE	GO:0004652	polynucleotide adenylyltransferase activity	0.3169	0.9524	-0.6354	E to B
BE	GO:0004502	kynurenine 3-monooxygenase activity	0.0219	0.6667	-0.6448	E to B
BE	GO:0004037	allantoicase activity	0.0656	0.7143	-0.6487	E to B
BE	GO:0004768	stearoyl-CoA 9-desaturase activity	0.1038	0.7619	-0.6581	E to B
BE	GO:0008603	cAMP-dependent protein kinase regulator activity	0.2295	0.9524	-0.7229	E to B
BE	GO:0004392	heme oxygenase (decyclizing) activity	0.1803	0.9048	-0.7244	E to B
BE	GO:0003870	5-aminolevulinate synthase	0.1639	0.9048	-0.7408	E to B

		activity				
BE	GO:0005544	calcium-dependent phospholipid binding	0.0164	0.7619	-0.7455	E to B
BE	GO:0008478	pyridoxal kinase activity	0.153	0.9048	-0.7518	E to B
BE	GO:0008441	3'(2'),5'-bisphosphate nucleotidase activity	0.1038	0.8571	-0.7533	E to B
BE	GO:0004622	lysophospholipase activity	0.1311	0.9048	-0.7736	E to B
BE	GO:0005516	calmodulin binding	0.0546	0.8571	-0.8025	E to B
BE	GO:0000062	fatty-acyl-CoA binding	0.1913	1	-0.8087	E to B
BE	GO:0004499	N,N-dimethylaniline monooxygenase activity	0.1803	1	-0.8197	E to B
BE	GO:0004408	holocytochrome-c synthase activity	0.0273	0.8571	-0.8298	E to B
BE	GO:0004198	calcium-dependent cysteine-type endopeptidase activity	0.0164	0.9524	-0.936	E to B
BE	GO:0008083	growth factor activity	0.0055	0.9524	-0.9469	E to B
BE	GO:0004610	phosphoacetylglucosamine mutase activity	0.0055	0.9524	-0.9469	E to B
BE	GO:0004001	adenosine kinase activity	0.0328	1	-0.9672	E to B
BE	GO:0004435	phosphatidylinositol phospholipase C activity	0.0273	1	-0.9727	E to B
BE	GO:0004571	mannosyl-oligosaccharide 1,2-alpha-mannosidase activity	0.0109	1	-0.9891	E to B
BE	GO:0004835	tubulin-tyrosine ligase activity	0.0109	1	-0.9891	E to B
BE	GO:0003873	6-phosphofructo-2-kinase activity	0.0109	1	-0.9891	E to B
BE	GO:0005484	SNAP receptor activity	0.0055	1	-0.9945	E to B
BE	GO:0004012	phospholipid-translocating ATPase activity	0.0055	1	-0.9945	E to B

TABLE S3: List of FSFs that were uniquely detected in the proteomes of the AE taxonomic group.

No.	Scop Id	FSF Id	FSF description
1	48140	a.94.1	Ribosomal protein L19 (L19e)
2	109993	a.222.1	VPS9 domain
3	53032	c.52.2	tRNA-intron endonuclease catalytic domain-like
4	54984	d.58.12	eEF-1beta-like
5	116742	a.60.14	eIF2alpha middle domain-like
6	118310	g.80.1	AN1-like Zinc finger
7	54575	d.29.1	Ribosomal protein L31e
8	55481	d.91.1	N-terminal domain of eukaryotic peptide chain release factor subunit 1, ERF1
9	89124	a.183.1	Nop domain
10	55003	d.58.16	PAP/Archaeal CCA-adding enzyme, C-terminal domain
11	55267	d.75.1	tRNA-intron endonuclease N-terminal domain-like
12	110993	d.58.51	eIF-2-alpha, C-terminal domain
13	69695	d.201.1	SRP19
14	82704	d.68.6	AlbA-like
15	48662	a.137.1	Ribosomal protein L39e
16	56741	e.15.1	Eukaryotic DNA topoisomerase I, N-terminal DNA-binding fragment
17	46950	a.5.6	Double-stranded DNA-binding domain
18	116820	a.4.15	Rps17e-like
19	75689	g.59.1	Zinc-binding domain of translation initiation factor 2 beta
20	143870	d.329.1	PF0523-like
21	88798	d.230.1	N-terminal, heterodimerisation domain of RBP7 (RpoE)
22	140726	a.253.1	AF0941-like
23	89895	d.235.1	FYSH domain
24	144210	g.41.16	Nop10-like SnoRNP
25	47157	a.23.4	Mitochondrial import receptor subunit Tom20
26	52042	c.9.2	Ribosomal protein L32e
27	111278	d.282.1	SSo0622-like
28	75399	d.211.2	Plakin repeat
29	103456	f.23.28	Preprotein translocase SecE subunit
30	63393	g.41.9	RNA polymerase subunits
31	55418	d.86.1	eIF4e-like
32	101576	b.132.1	Supernatant protein factor (SPF), C-terminal domain
33	141562	b.162.1	At5g01610-like
34	55287	d.78.1	RPB5-like RNA polymerase subunit
35	46924	a.4.11	RNA polymerase subunit RPB10
36	109728	a.5.8	Hypothetical protein AF0491, middle domain
37	100966	d.241.1	Translation initiation factor 2 beta, aIF2beta, N-terminal domain
38	88802	d.17.6	Pre-PUA domain

TABLE S4: List of terminal GOs that were uniquely detected in the functionomes of the AE taxonomic group.

No.	GO Id	GO description
1	GO:0000213	tRNA-intron endonuclease activity
2	GO:0004579	dolichyl-diphosphooligosaccharide-protein glycotransferase activity
3	GO:0004965	G-protein coupled GABA receptor activity
4	GO:0004164	diphthine synthase activity
5	GO:0030337	DNA polymerase processivity factor activity
6	GO:0017091	AU-rich element binding
7	GO:0030410	nicotianamine synthase activity
8	GO:0004776	succinate-CoA ligase (GDP-forming) activity
9	GO:0008466	glycogenin glucosyltransferase activity
10	GO:0003975	UDP-N-acetylglucosamine-dolichyl-phosphate N-acetylglucosaminephosphotransferase activity
11	GO:0004581	dolichyl-phosphate beta-glucosyltransferase activity

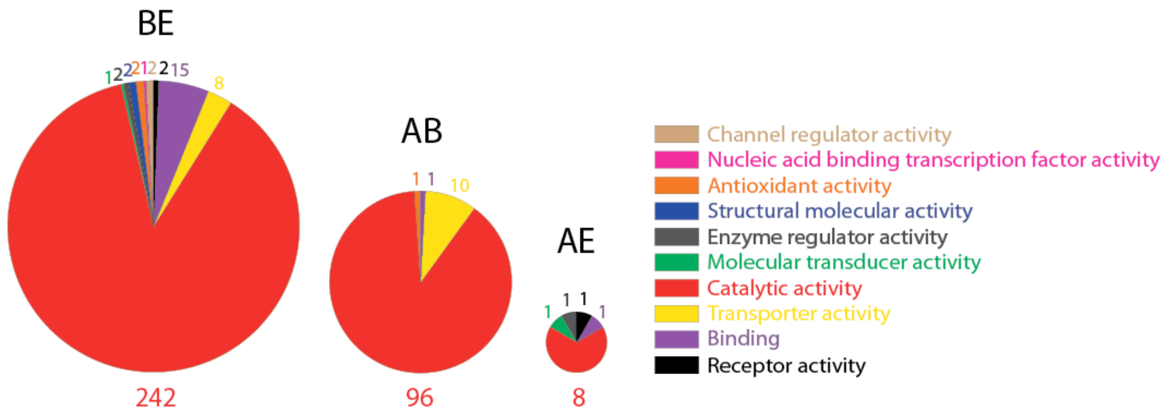

FIGURE S1: Pie charts displaying the distribution of terminal GOs in level-1 parent terms. Numbers indicate total number of terminal GOs annotated to each parent term category. Terms may be mapped to more than one parent.
